# Growth-Linked, Tissue-Specific Antioxidant Reprogramming During Natural Zn/Cu Bioaccumulation in the Pacific Oyster *Magallana gigas*

**DOI:** 10.3390/antiox15081039

**Published:** 2026-08-21

**Authors:** Bo-Wen Huang, Chen-Feng Liu, Mao-Le Wei, Xiang Zhang, Hui-Gang Kang, Kai-Jie Wang, Chang-Ming Bai

**Affiliations:** 1 State Key Laboratory of Mariculture Biobreeding and Sustainable Goods, Key Laboratory of Maricultural Organism Disease Control, Ministry of Agriculture, Yellow Sea Fisheries Research Institute, Chinese Academy of Fishery Sciences, Qingdao 266071, China; huangbw@ysfri.ac.cn (B.-W.H.); liucf9864@163.com (C.-F.L.); liuli000315@gmail.com (M.-L.W.); zhxiang1997@126.com (X.Z.); kanghuigang0123@163.com (H.-G.K.); wangkj0725@163.com (K.-J.W.); 2 Laboratory for Marine Fisheries Science and Food Production Processes, Qingdao National Laboratory for Marine Science and Technology, Qingdao 266237, China; 3 Shandong Technology Innovation Center of Oyster Seed Industry, Qingdao 266105, China

**Keywords:** growth, *Magallana gigas*, oxidative stress, transcriptomics, Zn/Cu bioaccumulation

## Abstract

Whether zinc (Zn) and copper (Cu) bioaccumulation in the Pacific oyster (*Magallana gigas*) reflects toxicological stress or is an incidental consequence of growth remains unclear. We cultured three commercial triploid *M. gigas* stocks for approximately one year, sampling gill and hepatopancreas at the start and end of this period, when Zn/Cu burden was naturally low and high, respectively. Pooled samples from both time points were profiled by whole-transcriptome sequencing, enzyme activity and oxidative damage assays, qPCR validation, and protein–protein interaction network analysis. Transcriptome-wide changes in both tissues tracked the culture period, but growth and Zn/Cu burden were too highly collinear (r = 0.92–0.98) to separate statistically. Critically, of the four metals measured (Zn, Cu, iron [Fe], and manganese [Mn]), only Zn and Cu increased with growth, whereas Fe and Mn did not, indicating metal-specific rather than generalized accumulation. Superoxide dismutase (SOD) activity and the transcript abundance of its copper/zinc isoform (Cu/Zn-SOD) increased with growth in both tissues, whereas catalase (CAT) activity was unchanged and glutathione peroxidase (GPX) activity rose only in gill. Malondialdehyde (MDA), a marker of oxidative damage, increased in both tissues. Gill mounted a broader response than hepatopancreas, including upregulation of KEAP1 alongside downregulation of detoxification, proteostasis, and ribosome-related genes. Stock-level qPCR further revealed stock-dependent regulation of antioxidant genes in hepatopancreas. Together, these results indicate that Zn/Cu bioaccumulation in *M. gigas* co-varies with growth in a metal-specific manner, consistent with cofactor demand for Cu/Zn-SOD. The accompanying oxidative and proteostatic changes therefore more plausibly reflect growth physiology than an independent pollutant signal.

## 1. Introduction

The Pacific oyster (*Magallana gigas*, formerly *Crassostrea gigas*) is among the most produced farmed aquatic animal species worldwide by tonnage [1]. As sessile filter feeders, oysters concentrate waterborne trace metals in their tissues, sometimes to levels several orders of magnitude above ambient seawater. This property has long made bivalves a preferred sentinel for coastal metal monitoring [2,3,4]. Such monitoring increasingly encompasses metals of industrial and nanotechnological concern, including silver (Ag), arsenic (As), and chromium (Cr). These co-occur with zinc (Zn) and copper (Cu) in oysters from Chinese aquaculture areas [3]. Zn and Cu are of particular interest among these metals. Both are essential cofactors for a range of metalloenzymes, including Cu/Zn superoxide dismutase (Cu/Zn-SOD) [5], carbonic anhydrase [6], and cytochrome c oxidase [7], so deficiency can impair cellular function almost as much as excess. At the same time, elevated tissue burdens of the same two metals are separately flagged as an ecological risk in other aquatic systems [8,9]. Tissue Zn/Cu accumulation alone therefore says little about whether an oyster is thriving. Biological significance depends on how the tissue uses these metals, not merely how much of them it retains [10].

One of the clearest links between Zn/Cu status and cellular physiology runs through the antioxidant defense system. Cu/Zn-SOD is among the best characterized metalloenzymes in aerobic biology. Copper cycles between oxidation states to dismutate the superoxide radical, while zinc, catalytically silent, stabilizes the active site structurally [5]. *M. gigas* carries at least two Cu/Zn-SOD family proteins: a canonical, catalytically active cytoplasmic form, and a second that has lost enzymatic activity while retaining strong Zn/Cu-binding capacity and a distinct developmental expression pattern. A broader genome-wide survey across four oyster species (*M. gigas*, *Crassostrea virginica*, *Magallana hongkongensis*, and *Saccostrea glomerata*) similarly found the Cu/Zn-SOD family expanded into multiple subgroups, with conservation in hepatopancreas and stress responsiveness. This suggests the gene family has diversified toward metal-binding as well as antioxidant roles more broadly in oysters [11,12]. Because SOD activity depends directly on cofactor supply, tissue Zn/Cu accumulation could plausibly reinforce this first line of defense. Downstream, catalase (CAT), glutathione peroxidase (GPX), the glutathione (GSH) system, and the Nrf2/Keap1 regulatory pair together determine whether reactive oxygen species (ROS) are cleared efficiently or allowed to accumulate and damage lipids. Such damage is commonly tracked via malondialdehyde (MDA) [13,14]. In *M. gigas*, the Nrf2/Keap1 pathway is confirmed at the genomic level, and its experimental activation produces markedly tissue-specific responses. Curcumin or tert-butylhydroquinone exposure induces glutathione-related defenses in gill but not in hepatopancreas of the same animal [15,16], a pattern also reported under prolonged dietary antioxidant supplementation [17]. Zn and Cu do not act independently on this system. Zinc co-exposure under Cu stress altered oxidative stress and immune-related gene expression in *M. gigas*, and a field survey of the congeneric *M. hongkongensis* found gill Cu/Zn burden correlating positively with lipid peroxidation and antioxidant enzyme activity, while metallothionein moved in the opposite direction [18,19]. Zinc alone likewise perturbs the *M. gigas* gill metabolome along oxidative-stress and energy-metabolism pathways [20]. This tissue asymmetry extends beyond enzymes. Metallothionein rises in gill, whereas hepatopancreas instead loses total protein content under the same exposure, indicating genuinely distinct metal-handling strategies rather than one response scaled differently between tissues [21]. Such divergence was documented in bivalves before the Nrf2/Keap1 pathway itself was characterized in mollusks [22]. A transcriptome survey of the related species *Crassostrea gasar* likewise treated gill and hepatopancreas as biologically distinct compartments when screening for metal-metabolism genes [23].

A separate line of evidence points to growth itself as a driver of oxidative burden. Across a wide range of taxa, including bivalves, elevated metabolic rate increases mitochondrial electron flux and, correspondingly, superoxide leakage, linking life-history strategy to oxidative stress and longevity [24]. Bivalve energy budgets are sensitive to trace-element exposure. The scope-for-growth literature lists Cu, Zn, and cadmium (Cd) alongside temperature, salinity, and dissolved oxygen among the factors shifting the balance between growth and maintenance [25,26]. Comparative transcriptomic and metabolomic work in oysters has shown that faster-growing lineages allocate relatively more resources to antioxidant gene expression while suppressing glycolysis [27]. Independent of metabolic rate, absolute filtration capacity in bivalves generally scales with body size, following a power-law relationship with shell dimensions and tissue mass in at least one other filter-feeding bivalve [28]. Larger, growing oysters would therefore be expected to filter more seawater in absolute terms and incidentally accumulate more of the Zn/Cu it contains [29]. A genetic component may also be at play. A genome-wide association study in *M. gigas* itself linked zinc-transporter and caveolin-mediated endocytosis genes directly to individual variation in Zn and Cu accumulation [30]. A separate genome-wide association study in a congeneric oyster linked zinc-transporter (ZIP) family loci to individual variation in Cd accumulation [31], and functional work on two candidates, *Zip1* and *Zip3*, showed that RNAi knockdown shifted Cd uptake in opposite directions [32]. Comparative work on genetically distinct lines of the eastern oyster (*C. virginica*) has similarly linked growth performance to distinct GO- and KEGG-enriched expression programs [33]. This raises the possibility that the stocks examined here differ not only in growth rate but also in how they regulate trace-metal handling. We therefore hypothesized that growth, metal accumulation, and antioxidant transcriptional regulation may be facets of a single underlying physiology rather than independent phenomena.

Despite this evidence, most studies of trace-metal oxidative stress in bivalves rely on short, researcher-controlled exposures at a fixed dose against unexposed controls. Transcriptomic studies of *M. gigas* gill have similarly focused on acute exposure to a single metal, typically Cd, rather than naturally accumulated Zn/Cu [15,16,17,18,34]. This design suits dose–response questions but cannot capture gradual accumulation over extended aquaculture, nor separate metal burden from the growth process it accompanies. One exception tracked toxic-metal accumulation alongside a full oxidative-stress biomarker panel across a natural growing season in the razor clam *Sinonovacula constricta*, finding that metal burden and several biomarkers rose together as the animals grew [35]. This work, however, remained at the biochemical level without transcriptomic analysis. Field surveys of wild *M. hongkongensis* populations across contamination gradients have similarly found correlations between tissue Cu/Zn burden and antioxidant enzyme activity [19], but they cannot separate a location effect from within-population, growth-linked accumulation. Season introduces a further confound. In the clam *Ruditapes decussatus*, oxidative-stress enzymes and lipid peroxidation at a single polluted site differed between summer and winter independently of metal burden, likely reflecting reproductive stage and food availability [36]. To our knowledge, no comparable study of natural, growth-period Zn/Cu accumulation in *M. gigas* has been paired with whole-transcriptome profiling in any bivalve species. Whether the tissue-specific antioxidant responses documented under controlled exposure also characterize this slower, naturally occurring accumulation, and what transcriptional program underlies them, remains an open question.

In this study, we followed three commercial triploid stocks of Pacific oysters (*M. gigas*), obtained from three independent hatcheries so that stock origin could be treated as a blocking factor, through approximately one year of commercial-scale culture in Sanggou Bay, China. We compared pooled tissue samples from the beginning and the end of this period, which corresponded to naturally lower and higher tissue Zn/Cu accumulation, respectively. We combined whole-transcriptome sequencing with gene ontology and pathway enrichment analysis, targeted enzyme activity and oxidative damage assays (SOD, CAT, GPX, MDA, GSH), qPCR validation of key genes (Cu/Zn-SOD, GPX1, KEAP1, CAT), and protein–protein interaction network analysis in gill and hepatopancreas. Our aim was to test whether naturally occurring Zn/Cu accumulation over a full growth cycle is accompanied by a coordinated, tissue-specific antioxidant response at both transcriptional and biochemical levels. In doing so, we sought to clarify the biological significance of metal bioaccumulation in this commercially important filter-feeding bivalve.

## 2. Materials and Methods

### 2.1. Oyster Collection

Triploid Pacific oysters (*M. gigas*) from three commercial stocks (designated N, Y, and R; arbitrary labels with no further meaning) were used in this study. The three stocks were produced in March 2023 by three major Chinese commercial hatcheries as triploid seed generated by tetraploid × diploid crosses. Oysters were cultured at Sanggou Bay, Weihai, Shandong Province, China, for approximately one year. Each stock was sampled at four time points across this period (230816, 231221, 240310, and 240720, given as YYMMDD), and gill and hepatopancreas tissue were collected from every individual. For each stock, sampling date, and tissue, ten oysters were pooled to form a single biological replicate, and three such replicates were prepared. Each pooled sample was divided into three portions: one for tissue metal content, one mixed with TRIzol reagent (Invitrogen, Carlsbad, CA, USA) for RNA extraction (used for both transcriptome sequencing and qPCR), and one reserved for antioxidant enzyme activity and oxidative stress measurements. All three replicates were used for metal content, enzyme activity, and qPCR measurements, whereas two of the three were used for transcriptome sequencing. Every portion was immediately frozen in liquid nitrogen and stored at −80 °C until analysis.

### 2.2. Tissue Metal Content and Growth Measurement

Tissue samples were weighed into digestion vessels and treated with 5 mL of concentrated nitric acid (guaranteed reagent grade; Merck, Darmstadt, Germany). Samples were digested under high pressure for 2 h and then diluted to a final volume of 50 mL before analysis. The Zn, Cu, iron (Fe), and manganese (Mn) contents were then determined by inductively coupled plasma optical emission spectrometry (ICP-OES; iCAP7200 Duo, Thermo Fisher Scientific, Waltham, MA, USA), following the Chinese national standard GB 5009.268-2016 [37]. Shell height, shell length, and total wet weight were measured directly with calipers and a balance at each sampling date. Three biological replicates were measured per stock and sampling date for Zn, Cu, Fe, and Mn (*n* = 9, pooled across the three stocks), whereas total wet weight was recorded individually for 10 of these oysters per stock and date (*n* = 30, pooled across the three stocks). Based on these measurements, samples from the first (230816) and last (240720) sampling dates, corresponding to lower and higher tissue Zn/Cu content, are hereafter designated the low- (L) and high- (H) accumulation groups.

### 2.3. Measurement of Oxidative Stress Indicators (OSIs)

The activities of superoxide dismutase (SOD), glutathione peroxidase (GPX), and catalase (CAT), together with the contents of malondialdehyde (MDA) and reduced glutathione (GSH), were used as OSIs and were measured in gill and hepatopancreas tissue collected at the first (230816) and last (240720) sampling dates. Measurements used the superoxide dismutase assay kit (A001-3-2), catalase assay kit (A007-1-1), glutathione peroxidase assay kit (A005-1-2), malondialdehyde assay kit (A003-1-2), and reduced glutathione assay kit (A006-2-1), respectively, following the manufacturer’s instructions (Nanjing Jiancheng Bioengineering Institute, Nanjing, China). Three biological replicates were measured per stock, tissue, and sampling date (*n* = 9, pooled across the three stocks).

### 2.4. Library Preparation and Sequencing

RNA purification, library preparation, and sequencing were performed by Novogene (Beijing, China). RNA integrity was assessed on a Bioanalyzer 2100 system (Agilent Technologies, Santa Clara, CA, USA) using the RNA Nano 6000 Assay Kit. Sequencing libraries were constructed from total RNA. mRNA was enriched using poly-T oligo-attached magnetic beads, fragmented, and reverse-transcribed into first- and second-strand cDNA, followed by end repair. After 3′ adenylation, adaptors with a hairpin-loop structure were ligated, and fragments of approximately 370–420 bp were size-selected. Libraries were amplified by PCR, purified, and quality-checked on the Bioanalyzer 2100 system before sequencing on an Illumina NovaSeq platform (Illumina, San Diego, CA, USA, 150 bp paired-end reads). Two tissues (gill and hepatopancreas) × two accumulation groups (H and L) were each represented by 3 stocks × 2 biological replicates, yielding 24 sequencing libraries in total. The three stocks were treated as pooled biological replicates within each tissue × group combination.

### 2.5. Read Processing and Differential Expression Analysis

Raw sequencing reads were quality-filtered using fastp to remove adapter sequences, reads containing a high proportion of ambiguous bases, poly(A) reads, and low-quality reads. Q20, Q30, and GC content were then calculated for the resulting clean data. Across the 24 sequencing libraries, clean reads per sample ranged from 41,049,250 to 54,530,336 (6.16–8.18 Gb clean data), with Q20 values of 98.42–99.15%, Q30 values of 95.47–97.56%, and GC content of 41.9–43.2%. Clean reads were aligned to the *M. gigas* reference genome (GCF_963853765.1) using HISAT2 (v2.0.5), with total mapping rates of 59.16–72.47% and unique mapping rates of 56.77–69.80% per sample. Gene-level read counts and expression values (FPKM) were quantified using featureCounts (v1.5.0-p3). Differential expression between the H and L accumulation groups was assessed separately for each tissue (Hg vs. Lg for gill; Hh vs. Lh for hepatopancreas) using the DESeq2 R package (v1.20.0), which models raw read counts under a negative binomial distribution. Resulting *p* values were adjusted using the Benjamini–Hochberg procedure. Genes were considered significantly differentially expressed at padj ≤ 0.05 and |log2FoldChange| ≥ 1.

### 2.6. Functional Enrichment Analysis

Over-representation analysis (ORA) of gene ontology (GO) terms and KEGG pathways was performed on differentially expressed genes (padj ≤ 0.05, |log2FoldChange| ≥ 1), separately for upregulated and downregulated gene sets in each tissue comparison. Enrichment was tested using a hypergeometric test with Benjamini–Hochberg correction (significance threshold: padj ≤ 0.05). Gene set enrichment analysis (GSEA) was performed on the full ranked gene list (ranked by the DESeq2 test statistic/signal-to-noise metric) against the GO and KEGG gene set collections, using GSEA as implemented through Novogene’s bioinformatics analysis platform (Beijing, China). Gene sets were considered significant at a nominal *p* < 0.05 and FDR q < 0.25, following standard GSEA convention. To assess whether the relative detection rates of the two methods depended on this convention, the ORA–GSEA comparison was repeated at stricter GSEA thresholds (FDR q < 0.10 and q < 0.05). GO terms related to oxidative stress and antioxidant defense were additionally selected *a priori* for detailed presentation. Each term’s rank among all significant terms in its direction of change was reported explicitly, to distinguish this hypothesis-driven selection from an unbiased top-ranked-term summary.

### 2.7. Principal Component Analysis and Correlation with Growth and Metal Content

Principal component analysis (PCA) was performed on the whole-transcriptome expression matrix across all 24 sequenced samples, as part of the standard bioinformatic pipeline provided by Novogene (Beijing, China), using R (v3.5.0) with visualization via the ggplot2 package. Sample scores on PC2, the component separating the H and L accumulation groups, were then used for downstream correlation analyses. Pearson correlation was calculated between PC2 scores and each of five sample-level variables: shell height, shell length, total weight, and tissue Zn and Cu content. Because Zn and Cu content were highly collinear with growth traits, partial correlation between metal content and PC2 was additionally computed after statistically adjusting for total weight (and, separately, for all three growth traits jointly), using a residualization approach. PC2 and the metal variable were each regressed on the growth covariate(s) by ordinary least squares, and the Pearson correlation was calculated between the resulting residuals.

### 2.8. Protein–Protein Interaction Network Analysis

Twenty genes representing six functional categories relevant to antioxidant defense and redox homeostasis (ROS dismutation, H_2_O_2_/peroxide clearance, GSH synthesis and recycling, phase-II detoxification, the thioredoxin system, and a master regulator) were mapped to their human orthologs and queried against the STRING database (v12; minimum combined interaction confidence score ≥ 0.4). Network visualization used an identical node layout for both tissues, with node fill representing the oyster log2FoldChange (H vs. L) for that gene in the corresponding tissue comparison.

### 2.9. cDNA Synthesis and qPCR Analysis

Total RNA, extracted as described in Section 2.1 (H and L accumulation groups; three biological replicates per stock [N, Y, R] per tissue per group), was reverse-transcribed into first-strand cDNA using the PrimeScript RT reagent Kit with gDNA Eraser (TaKaRa, Dalian, China), following the manufacturer’s protocol. qPCR was performed on an ABI 7500 Real-Time PCR System (Applied Biosystems, Foster City, CA, USA) with primers specific to Cu/Zn-SOD, GPX1, KEAP1, and CAT. EF1-α was amplified with a separate primer pair as the endogenous control. Primers used for qPCR are listed in Table S1. Each reaction was carried out in a final volume of 20 μL, containing 10 μL of 2× SYBR Green Mix (TaKaRa, Dalian, China), 1 μL of each primer (10 μM), and 8 μL of cDNA diluted 1:100. The amplification protocol consisted of an initial denaturation at 95 °C for 1 min, followed by 40 cycles of 95 °C for 5 s and 60 °C for 34 s. Relative expression of each target gene was calculated using the 2^−ΔΔCt^ method, with the low-accumulation (L) group serving as the calibrator within each stock × tissue combination. Three biological replicates were measured per stock per tissue per group (*n* = 9, pooled across the three stocks).

### 2.10. Statistical Analysis

All statistical analyses were performed in Python (v3.12) using scipy.stats and NumPy, except where noted. For datasets in which three genetically distinct stocks (N, Y, R) were each represented by multiple biological replicates (tissue metal/growth data, enzyme activity/oxidative damage markers, and qPCR data), a two-way ANOVA was used, with the biological effect of interest (sampling date or accumulation group) as the main effect and stock as a blocking factor. This design avoided confounding stock-level baseline differences with the effect of interest. It was chosen over a naive pooled t-test, which risks conflating stock and treatment effects, and over a paired test on stock-level means alone, which is statistically valid but underpowered given only three stocks. A stock × group (or stock × date) interaction term was additionally tested for each variable, to assess whether the direction and/or magnitude of change was consistent across stocks. Where the interaction was significant, per-stock values were reported explicitly rather than only a pooled summary statistic. For qPCR data specifically, ANOVA was performed on −ΔCt values (log2 scale), whereas the fold-change values reported in the text and figures (2^−ΔΔCt^, with mean ± SEM/SD) were presented on the linear scale for interpretability, following standard qPCR reporting convention. Because fold-change data of this kind are approximately log-normally distributed, the linear-scale SD/SEM values reported can appear large relative to the mean. The significance tests themselves should therefore be understood as based on the log-scale values rather than on the linear-scale descriptive statistics. RNA-seq differential expression, GO/KEGG ORA, and GSEA statistics were computed as described in Section 2.5 and Section 2.6, using their respective established statistical frameworks. Correlation and partial correlation analyses (Section 2.7) used Pearson’s r with two-tailed significance testing. Spearman’s rank correlation was additionally used for the association between MDA and enzyme activity ratios (Section 3.2), and for the concordance between RNA-seq and qPCR log2 fold changes (Section 3.7). A significance threshold of α = 0.05 was used throughout unless otherwise specified (e.g., the FDR q < 0.25 convention for GSEA).

## 3. Results

### 3.1. Zinc and Copper Accumulate Progressively in Oyster Tissue Alongside Growth, While Iron and Manganese Remain Stable

**Figure 1:**
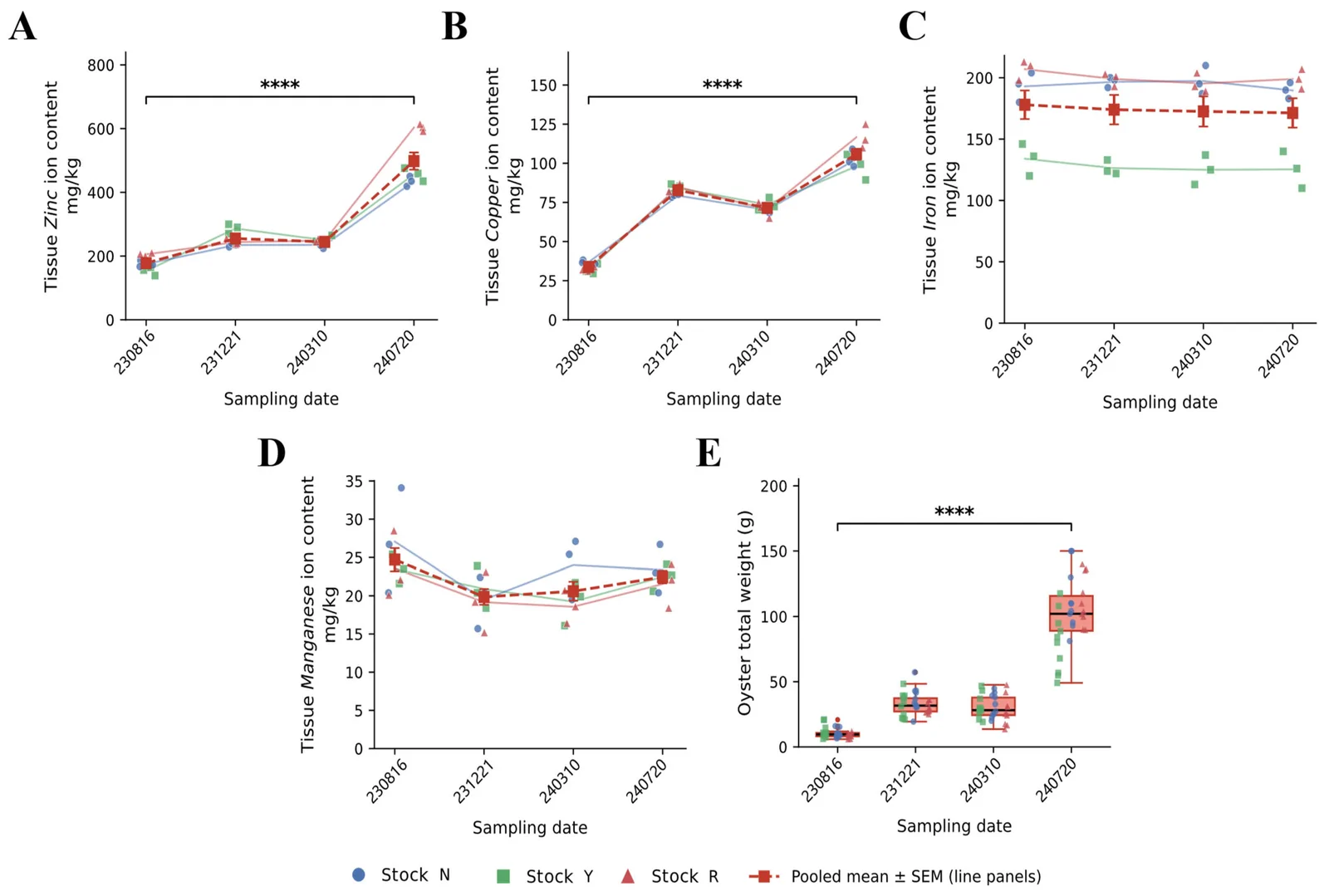
Tissue metal ion content and oyster growth across the culture period. (**A**) Tissue zinc content, (**B**) tissue copper content, (**C**) tissue iron content, (**D**) tissue manganese content (mg/kg), and (**E**) oyster total wet weight (g), at four sampling dates (230816, 231221, 240310, 240720). Colored symbols denote individual stock means (Stock N, blue circles; Y, green squares; R, red triangles); the red dashed line with square markers shows the pooled mean ± SEM across all three stocks (*n* = 9 per date for (**A**–**D**); *n* = 30 per date for (**E**)). Boxplots in (**E**) show the pooled distribution (median, interquartile range, whiskers) with individual stock data points overlaid. Statistical comparisons (first vs. last sampling date) used a two-way ANOVA with sampling date and stock as factors; **** *p* < 0.0001.

To characterize trace-metal handling over the culture period, we quantified tissue Zn, Cu, Fe, and Mn content together with oyster total wet weight at four sampling dates spanning approximately one year (230816, 231221, 240310, 240720; Figure 1). Tissue Zn content increased from 177.9 ± 24.2 mg/kg to 498.2 ± 80.6 mg/kg, and Cu content increased from 33.8 ± 2.9 mg/kg to 105.8 ± 10.5 mg/kg, between the first and last sampling dates (*p* < 0.0001; Figure 1A,B). Total wet weight increased from 10.4 ± 3.8 g to 101.8 ± 26.3 g over the same interval (*p* < 0.0001; Figure 1E). By contrast, tissue Fe content (178.0 ± 35.0 to 171.3 ± 35.9 mg/kg) and Mn content (24.7 ± 4.5 to 22.5 ± 2.5 mg/kg) did not change significantly across the culture period (*p* > 0.05; Figure 1C,D), indicating that Zn and Cu accumulation reflects a specific bioaccumulation process rather than a generalized increase in trace-metal loading. All three stocks (N, Y, R) showed the same direction of change for all five variables; however, a two-way ANOVA including stock as a factor revealed a significant stock × date interaction for Zn, Cu, and total weight (F = 45.20, *p* = 6.5 × 10^−12^; F = 4.64, *p* = 0.0029; F = 5.79, *p* < 0.0001, respectively), reflecting stock-dependent differences in the rate, but not the direction, of change.

### 3.2. Antioxidant Enzyme Activity and Lipid Peroxidation Increase with Culture Duration, with Tissue-Specific Glutathione Peroxidase Induction

**Figure 2:**
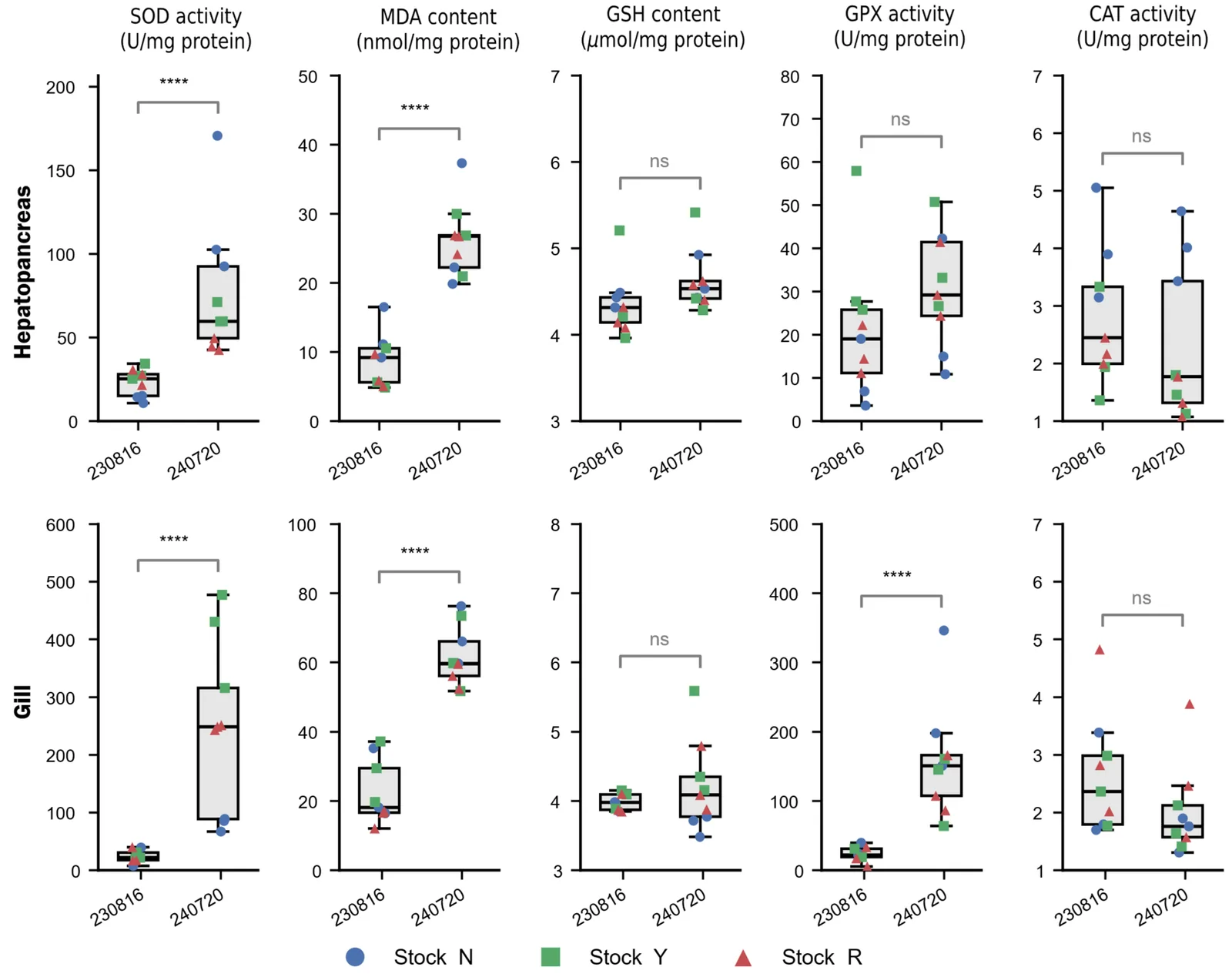
Antioxidant enzyme activity and oxidative damage markers in hepatopancreas and gill before and after culture. Superoxide dismutase (SOD) activity, malondialdehyde (MDA) content, glutathione (GSH) content, glutathione peroxidase (GPX) activity, and catalase (CAT) activity in hepatopancreas (top row) and gill (bottom row) at the first (230816) and last (240720) sampling dates. Boxplots show the pooled distribution across three stocks (Stock N, blue circles; Y, green squares; R, red triangles; *n* = 9 per group), with individual stock data points overlaid. Statistical comparisons used a two-way ANOVA with sampling date as the effect of interest and stock as a blocking factor; **** *p* < 0.0001, ns *p* ≥ 0.05.

We next measured SOD, GPX, and CAT activity together with MDA and GSH content in hepatopancreas and gill at the first and last sampling dates (Figure 2; *n* = 9 per group, pooled across three stocks). Statistical significance was assessed by two-way ANOVA with sampling date as the effect of interest and stock (N, Y, R) as a blocking factor, since the three stocks represent distinct genetic backgrounds rather than technical replicates. SOD activity increased from 23.0 ± 8.1 to 77.0 ± 40.8 U/mg protein in hepatopancreas (*p* < 0.0001) and from 24.0 ± 10.7 to 245.4 ± 147.9 U/mg protein in gill (*p* < 0.0001). MDA content increased from 8.7 ± 3.8 to 26.1 ± 5.3 nmol/mg protein in hepatopancreas and from 22.5 ± 9.1 to 61.7 ± 8.6 nmol/mg protein in gill (both *p* < 0.0001). GPX activity increased from 23.5 ± 10.1 to 158.4 ± 82.0 U/mg protein in gill (*p* < 0.0001) but did not change significantly in hepatopancreas (21.0 ± 16.1 to 30.4 ± 13.0 U/mg protein, *p* = 0.14). CAT activity did not change significantly in either tissue (hepatopancreas: 2.82 ± 1.15 to 2.29 ± 1.36 U/mg protein, *p* = 0.12; gill: 2.63 ± 1.02 to 2.01 ± 0.79 U/mg protein, *p* = 0.17). GSH content likewise did not change significantly in either tissue (hepatopancreas: 4.35 ± 0.36 to 4.62 ± 0.35 μmol/mg protein, *p* = 0.17; gill: 3.98 ± 0.12 to 4.20 ± 0.65 μmol/mg protein, *p* = 0.24). A significant stock × date interaction was detected for SOD in both tissues (*p* < 0.01); no significant interaction was detected for MDA, GSH, GPX, or CAT in either tissue (*p* > 0.05). MDA correlated positively with the SOD/CAT activity ratio when both sampling dates were combined (Spearman ρ = 0.67 in gill, 0.72 in hepatopancreas; *p* < 0.01), but not within either sampling date (all *p* > 0.25). The SOD/GPX ratio showed no correlation with MDA in either tissue (Table S2).

### 3.3. Global Transcriptomic Reprogramming Tracks Growth but Not Metal Accumulation Independently

**Figure 3:**
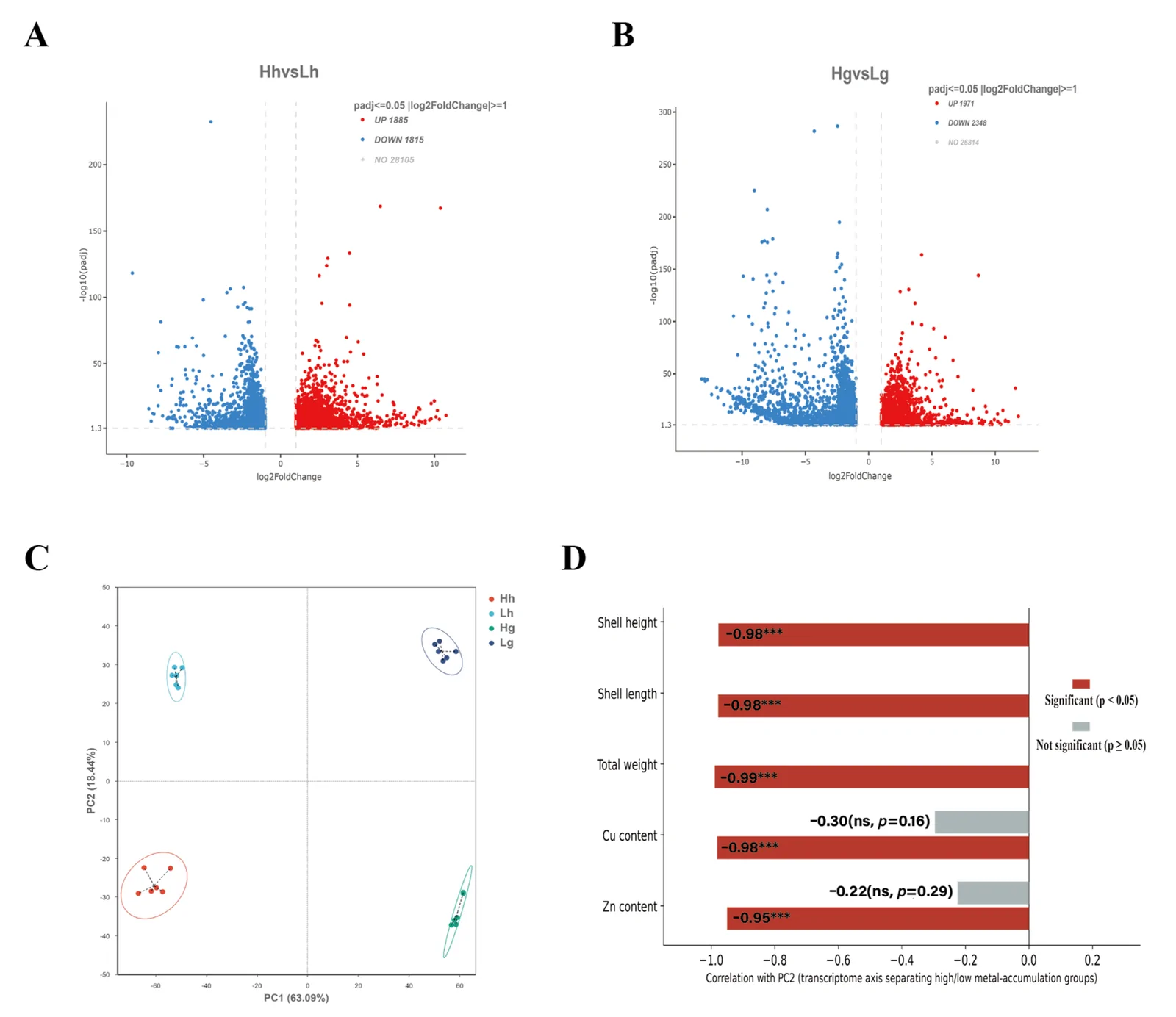
Global transcriptomic landscape across tissue and metal-accumulation groups. (**A**,**B**) Volcano plots of differentially expressed genes for the hepatopancreas comparison (Hh vs. Lh) and the gill comparison (Hg vs. Lg), padj ≤ 0.05 and |log2FoldChange| ≥ 1 (dashed lines). (**C**) Principal component analysis (PCA) of all samples, colored by tissue and metal-accumulation group (Hh, Lh, Hg, Lg). (**D**) Pearson correlations of growth traits (shell height, shell length, total weight) and tissue metal content (Cu, Zn) with PC2 (*n* = 24). For each variable, the first bar shows the zero-order correlation with PC2. For Cu and Zn content, a second bar shows the partial correlation with PC2 after adjusting for total weight; red indicates significant (*** *p* < 0.001), gray indicates not significant (ns *p* ≥ 0.05).

RNA sequencing identified 1885 upregulated and 1815 downregulated genes in hepatopancreas (Hh vs. Lh) and 1971 upregulated and 2348 downregulated genes in gill (Hg vs. Lg) (padj ≤ 0.05, |log2FoldChange| ≥ 1; Figure 3A,B; Table S3 and Table S4). PCA separated all samples primarily by tissue (PC1, 63.1% of variance) and secondarily by metal-accumulation group within each tissue (PC2, 18.4% of variance; Figure 3C). Growth traits (shell height, shell length, total weight) and tissue Zn and Cu content were all significantly correlated with PC2 at the zero-order level (r = −0.95 to −0.99, *p* < 0.0001; Figure 3D). Because Zn and Cu content were highly collinear with growth (r = 0.92–0.98), we additionally computed the partial correlation of Zn and Cu with PC2 after statistically adjusting for total weight; this partial correlation was r = −0.22 (*p* = 0.29) for Zn and r = −0.30 (*p* = 0.16) for Cu, whereas growth traits remained significantly correlated with PC2 independent of this adjustment (Figure 3D). Given that only two sampling groups were compared and that growth and metal content were highly collinear, this partial correlation is best read as a supporting observation rather than as decisive evidence of independent effects.

### 3.4. Key Antioxidant and Redox-Homeostasis Genes Show Coordinated Upregulation of Cu/Zn-SOD and KEAP1 Alongside Broad Downregulation of GSH-Cycling and Detoxification Genes

**Figure 4:**
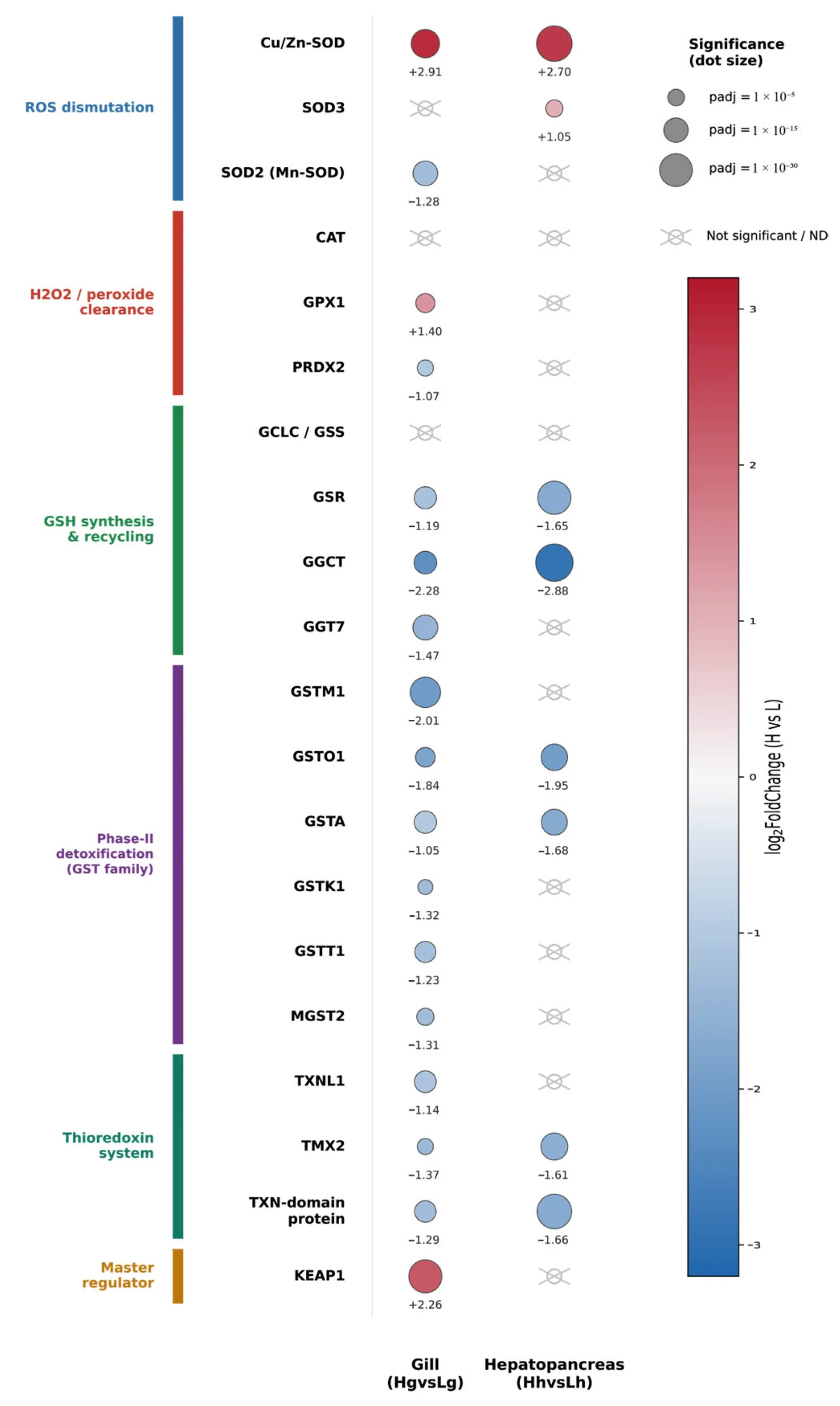
Transcriptional landscape of key antioxidant and redox-homeostasis genes. Bubble plot of log2FoldChange (dot color) and statistical significance (dot size, based on padj) for 20 genes across six functional categories (ROS dismutation, H_2_O_2_/peroxide clearance, GSH synthesis and recycling, phase-II detoxification (GST family), the thioredoxin system, and a master regulator (*KEAP1*)), shown separately for gill (Hg vs. Lg) and hepatopancreas (Hh vs. Lh). Crossed-out circles denote genes not significantly differentially expressed (padj ≥ 0.05 or |log2FoldChange| < 1) in that comparison. No transcript-level result is available for *CAT*, as no catalase gene was present in the annotation used for read quantification.

Among 20 genes spanning six functional categories, *Cu/Zn-SOD* was significantly upregulated in both gill (log2FC = 2.91) and hepatopancreas (log2FC = 2.70) (Figure 4). *KEAP1* was significantly upregulated in gill (log2FC = 2.26) but not detected as significant in hepatopancreas. *GPX1* was significantly upregulated in gill only (log2FC = 1.40). *CAT* was not represented in the annotation used for read quantification and could not be assessed at the transcript level. Genes involved in GSH synthesis and recycling (*GSR*, *GGCT*, *GGT7*) and in phase-II detoxification (the GST family: *GSTM1*, *GSTO1*, *GSTA*, *GSTK1*, *GSTT1*, *MGST2*) were predominantly downregulated in gill; *GSR*, *GGCT*, *GSTO1*, and *GSTA* were downregulated in both tissues (e.g., *GGCT*: log2FC = −2.28 in gill, −2.88 in hepatopancreas). Thioredoxin-system genes (*TXNL1*, *TMX2*, and a TXN-domain-containing gene) were downregulated mainly in gill, with *TMX2* and the TXN-domain gene also downregulated in hepatopancreas. Mapping these 20 genes to human orthologs in STRING (v12, confidence ≥ 0.4) resolved 18 of the 20 to a human ortholog (*GPX1* and *GSTT1* returned no match); using an identical network layout for both tissues, 14 of 18 nodes showed a significant oyster log2FoldChange in gill, compared with 7 of 18 in hepatopancreas (Figure S1A,B; Table S5).

### 3.5. Pathway-Level Enrichment Reveals Systemic Downregulation of Ribosome Biogenesis and Proteostasis Machinery Alongside Upregulated Immune/Lysosomal and Receptor-Signaling Pathways

**Figure 5:**
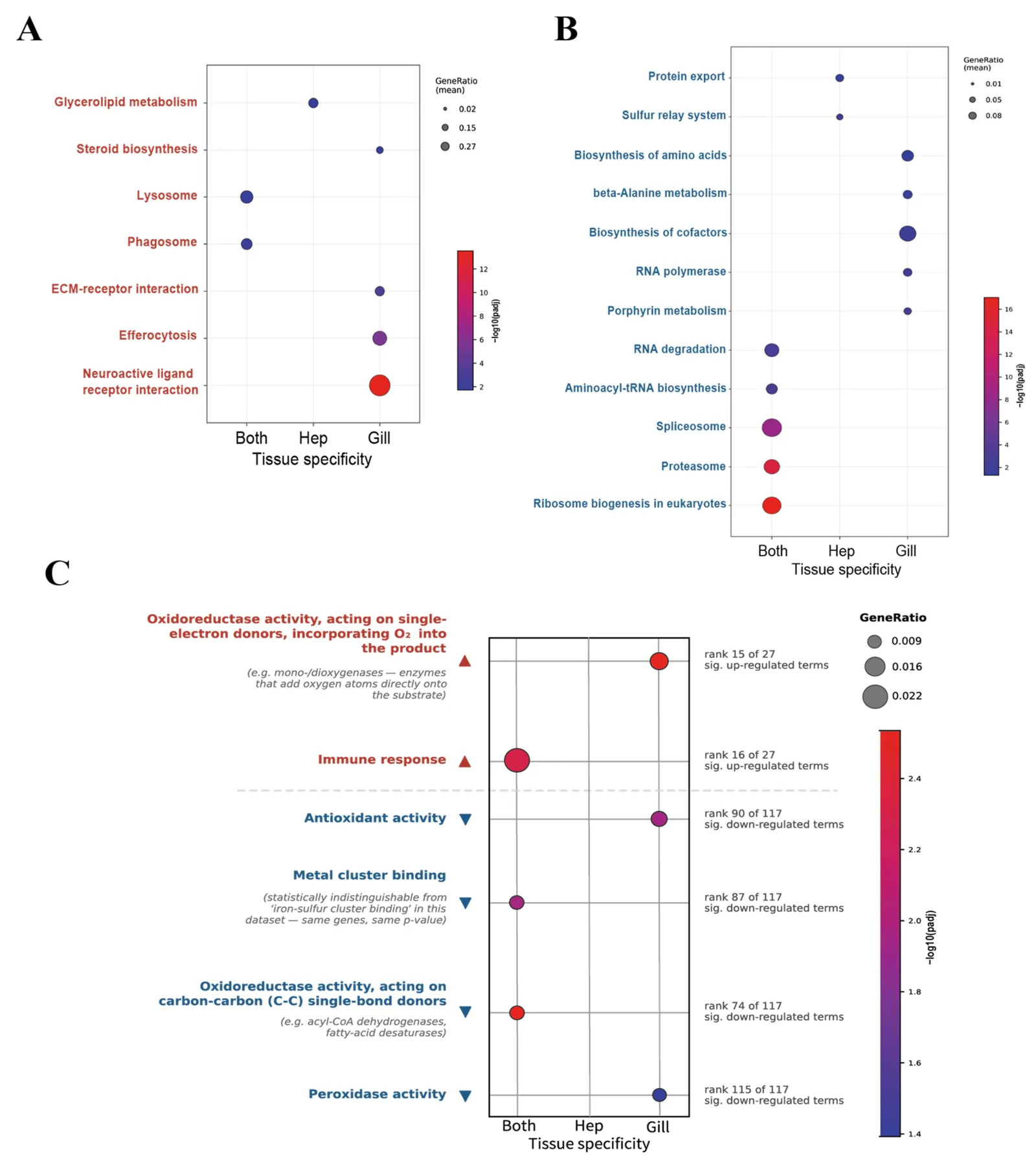
Pathway- and function-level enrichment evidence across tissues. (**A**,**B**) Upregulated and downregulated KEGG pathways identified by ORA (padj ≤ 0.05), classified by tissue specificity (Both, Hep-only, or Gill-only); dot size reflects the mean gene ratio, color reflects −log10(padj). (**C**) Selected GO terms related to oxidative stress and antioxidant defense, chosen *a priori* rather than by significance rank; the rank of each term among all significant terms for its direction (up, *n* = 27; down, *n* = 117) is shown alongside a plain-language gloss of GO terminology.

Over-representation analysis (ORA, Table S6) identified seven significantly upregulated KEGG pathways (padj ≤ 0.05): Lysosome and Phagosome (both tissues); neuroactive ligand–receptor interaction, ECM–receptor interaction, Efferocytosis, and Steroid biosynthesis (gill); and Glycerolipid metabolism (hepatopancreas) (Figure 5A). Twelve KEGG pathways were significantly downregulated, including Ribosome biogenesis in eukaryotes, Proteasome, Spliceosome, Aminoacyl-tRNA biosynthesis, and RNA degradation (both tissues), Biosynthesis of amino acids and Biosynthesis of cofactors (gill), and Protein export and Sulfur relay system (hepatopancreas) (Figure 5B). Ranking all significant GO terms by adjusted *p*-value in each tissue showed that protein ubiquitination and ubiquitin-protein transferase activity were the most significantly upregulated GO terms in both gill and hepatopancreas, while proteasome complex, ribosome biogenesis, and RNA-binding/translation-related terms were consistently among the most significantly downregulated GO terms in both tissues (Figure S2). Among GO terms selected *a priori* for relevance to oxidative stress and antioxidant defense, antioxidant activity and peroxidase activity were significantly downregulated in gill (ranked 90th and 115th of 117 significant downregulated terms); oxidoreductase activity acting on carbon–carbon single-bond donors and metal cluster binding were significantly downregulated in both tissues (ranked 74th and 87th); and immune response and an oxidoreductase term were significantly upregulated (ranked 16th and 15th of 27 significant upregulated terms) (Figure 5C).

### 3.6. GSEA Captures Coordinated Oxidative-Stress Pathway Signatures That Over-Representation Analysis Alone Misses

**Figure 6:**
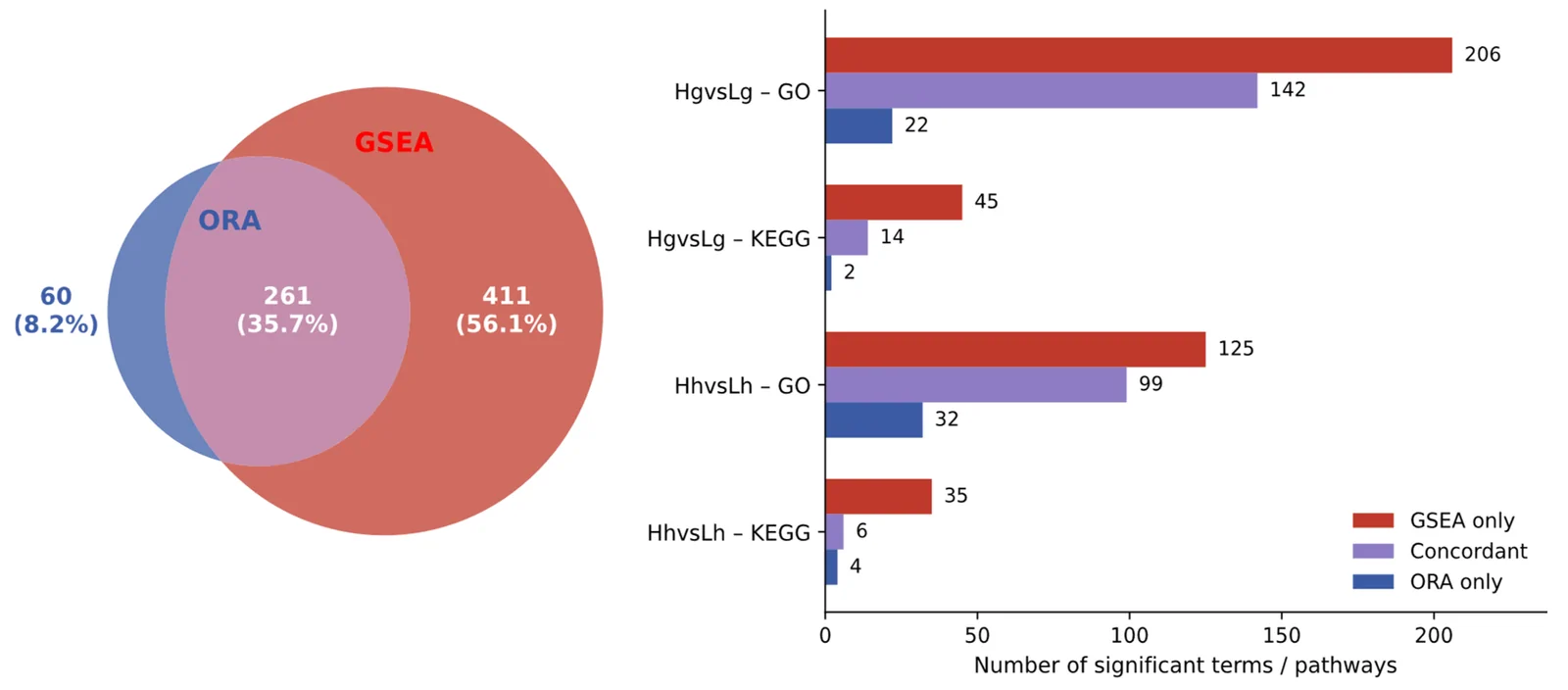
Concordance between ORA and GSEA. Area-proportional Venn diagram of significant GO/KEGG terms by ORA (padj ≤ 0.05) and GSEA (NOM *p* < 0.05, FDR q < 0.25), combined across tissues and ontologies, with the same breakdown shown by tissue × ontology (**right**).

Of 732 GO/KEGG terms reaching significance by ORA and/or GSEA across both tissue comparisons and ontologies, 261 (35.7%) were significant by both methods with concordant direction, 411 (56.1%) were significant by GSEA only, and 60 (8.2%) were significant by ORA only (Figure 6, Table S7). Three KEGG pathways (GLUTATHIONE_METABOLISM, PEROXISOME, and METABOLISM_OF_XENOBIOTICS_BY_CYTOCHROME_P450; gene-set identifiers as given in MSigDB) reached GSEA significance in both tissues but not by ORA in either tissue: in gill, NES = −1.77, −1.69, −1.85 (FDR q = 0.028, 0.038, 0.029); and in hepatopancreas, NES = −1.55, −1.60, −1.49 (FDR q = 0.085, 0.065, 0.11), all meeting the FDR q < 0.25 significance threshold defined in Section 2.6 (Figure S3). Because the FDR q < 0.25 convention used for GSEA is more permissive than the padj ≤ 0.05 threshold applied to ORA, we repeated this comparison at stricter GSEA thresholds (Table S8). GSEA-only terms remained the largest category at FDR q < 0.10 (285 of 606, 47.0%), whereas at q < 0.05 ORA-only terms became more numerous (207 of 472, 43.9%) than GSEA-only terms (151, 32.0%). The three pathways above all remained significant in gill at q < 0.05, while in hepatopancreas two met q < 0.10 and none met q < 0.05.

### 3.7. qPCR Quantification of Cu/Zn-SOD, GPX1, KEAP1, and CAT Expression

**Figure 7:**
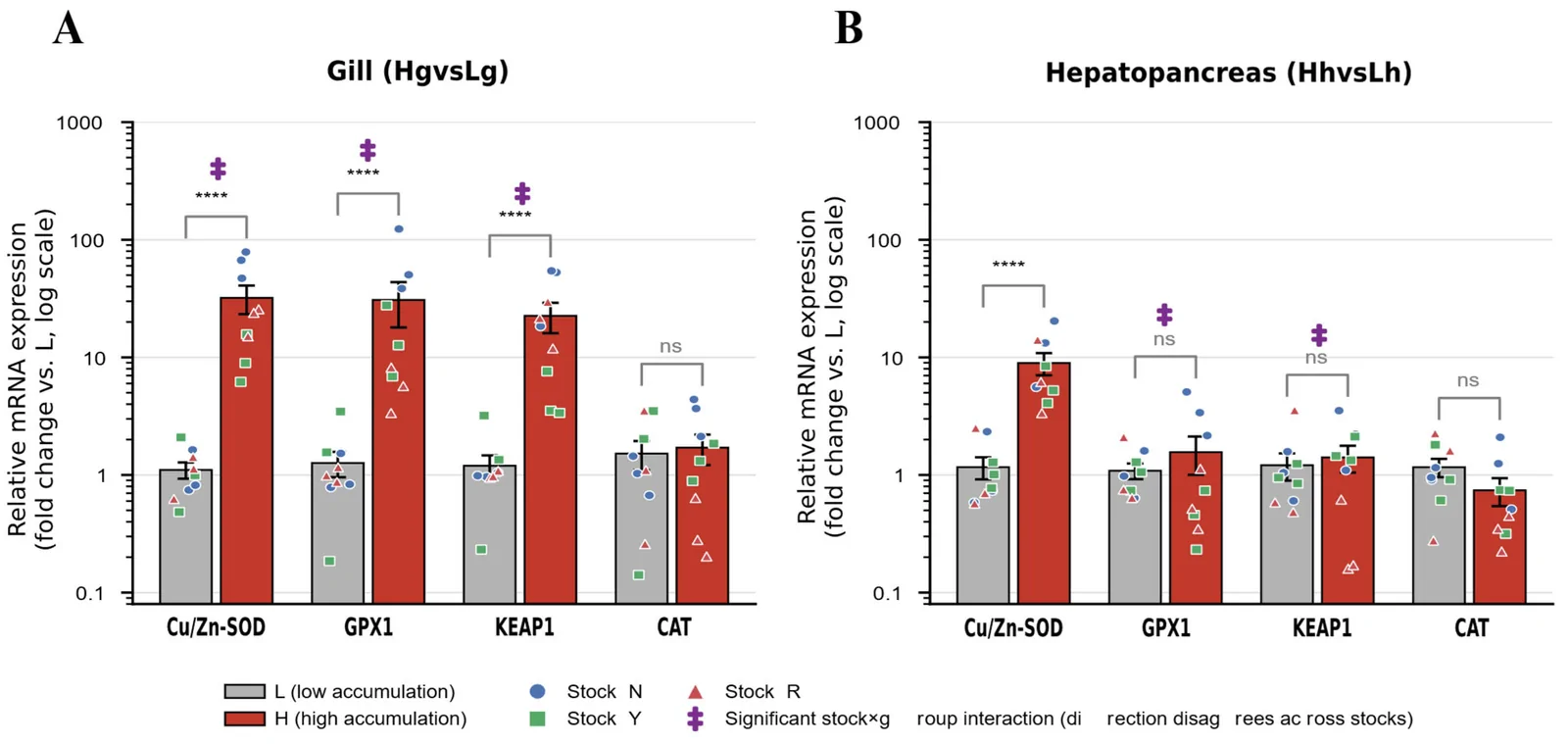
qPCR quantification of *Cu/Zn-SOD*, *GPX1*, *KEAP1*, and *CAT* expression by tissue and stock. Relative expression by the 2^−ΔΔCt^ method in (**A**) gill (Hg vs. Lg) and (**B**) hepatopancreas (Hh vs. Lh), comparing low- (L) and high- (H) accumulation groups. Bars show mean fold change ± SEM on a log-scale y-axis (*n* = 9 per group, pooled across three stocks); individual stock data points are overlaid (Stock N, blue circles; Y, green squares; R, red triangles). Statistical comparisons used a two-way ANOVA on −ΔCt with Group as the effect of interest and Stock as a blocking factor; **** *p* < 0.0001, ns *p* ≥ 0.05 (Group main effect); ‡ denotes a significant Stock × Group interaction (*p* < 0.05).

Relative expression of *Cu/Zn-SOD*, *GPX1*, *KEAP1*, and *CAT* was quantified by qPCR (Figure 7A,B). In gill (Figure 7A), *Cu/Zn-SOD* increased from 1.10 ± 0.17-fold (L) to 32.05 ± 8.74-fold (H) (*p* < 0.0001); *GPX1* increased from 1.26 ± 0.31-fold to 30.80 ± 12.84-fold (*p* < 0.0001); and *KEAP1* increased from 1.20 ± 0.27-fold to 22.57 ± 6.53-fold (*p* < 0.0001). *CAT* did not change significantly in gill (1.52 ± 0.42-fold to 1.71 ± 0.49-fold, *p* = 0.81). In hepatopancreas (Figure 7B), *Cu/Zn-SOD* increased from 1.17 ± 0.25-fold to 8.95 ± 1.91-fold (*p* < 0.0001). *GPX1* and *KEAP1* did not change significantly at the pooled level (*GPX1*: 1.09 ± 0.17-fold to 1.56 ± 0.56-fold, *p* = 0.81; *KEAP1*: 1.21 ± 0.31-fold to 1.41 ± 0.36-fold, *p* = 0.85). *CAT* likewise did not change significantly in hepatopancreas (1.16 ± 0.21-fold to 0.74 ± 0.20-fold, *p* = 0.10).

A significant stock × group interaction was detected for *GPX1* and *KEAP1* in hepatopancreas (*p* = 0.0098 and *p* = 0.031, respectively; Figure 7B). For *GPX1*, Stock N increased to 3.55 ± 0.85-fold of baseline, while Stock Y decreased to 0.48 ± 0.15-fold of baseline and Stock R decreased to 0.66 ± 0.24-fold of baseline. For *KEAP1*, Stock N increased to 2.28 ± 0.70-fold of baseline and Stock Y increased to 1.63 ± 0.24-fold of baseline, while Stock R decreased to 0.32 ± 0.15-fold of baseline. A significant stock × group interaction was also detected for *Cu/Zn-SOD* and *GPX1* in gill (*p* = 0.013 and *p* = 0.049, respectively; Figure 7A), where all three stocks changed in the same direction (per-stock values in Supplementary Table S9); no significant stock × group interaction was detected for *CAT* in either tissue (gill *p* = 0.15; hepatopancreas *p* = 0.30). Across the six gene × tissue pairs for which both measurements were available, RNA-seq and qPCR log2 fold changes were positively correlated (Pearson r = 0.85, *p* = 0.033; Spearman ρ = 0.75, *p* = 0.084; Figure S4), with a fitted slope of 1.47, indicating consistent direction and ranking but a systematically larger dynamic range for qPCR.

## 4. Discussion

Growth traits (shell height, shell length, and total weight) remained correlated with the transcriptome-wide axis separating early- and late-culture samples after adjusting for their collinearity with Zn/Cu content. Zn and Cu content, by contrast, did not survive the equivalent adjustment for growth. This partial correlation is only suggestive; however, since growth and metal content are too highly collinear over this period to be cleanly separated. The more decisive evidence is metal specificity. Of the four metals we measured, Fe and Mn did not increase over the culture period at all, whereas Zn and Cu did. This selectivity is notable because absolute filtration capacity in bivalves generally scales with body size and would be expected to raise tissue levels of any suspended element as the animal grows [28]. Growth, in other words, tracked two specific metals rather than trace-metal loading in general, which is more consistent with selective retention than with passive filtration. Indeed, under a biodynamic view, rapid growth tends to dilute tissue metal concentrations [29]. That Zn and Cu concentrations nonetheless rose severalfold while Fe and Mn remained flat is difficult to explain by passive, filtration-driven intake alone. A plausible basis for this selectivity is metabolic demand: sustained growth carries a real energetic cost through protein turnover [38]. Zn and Cu, unlike Fe and Mn, are the direct catalytic cofactors of Cu/Zn-SOD [5], the one enzyme among those we measured with a specific requirement for these two metals. Fe and Mn are themselves cofactors for other enzymes, including Mn-SOD and catalase, and the absence of any change in CAT activity or *CAT* expression measured by qPCR across the same samples is consistent with no comparable rise in demand for these two metals over the same period. This selective-retention account nonetheless remains a hypothesis: direct confirmation would require measurements we did not perform, such as metallothionein quantification, subcellular metal fractionation, or biodynamic flux assays across the growth cycle.

This account predicts that Cu/Zn-SOD itself should rise with growth. It did: the Cu/Zn-SOD transcript rose sharply in both tissues, accompanied by a parallel increase in total SOD activity. The qPCR fold-changes were considerably larger than the RNA-seq log2FoldChange values on their own would suggest, consistent with the more compressed dynamic range typical of RNA-seq. The two methods nonetheless agreed in direction and in the relative ranking of genes (Figure S4). SOD did not act alone; however, the rest of the antioxidant cascade did not move with it uniformly. A dissociation between SOD and CAT activity under metal exposure is a recognized pattern in bivalve ecotoxicology. In *Mytilus galloprovincialis* sampled across a heavy-metal contamination gradient, the two enzymes did not move together, and this asymmetry itself served as a pollution biomarker [39]. A related pattern was recently reported in *M. gigas* hemocytes under controlled copper exposure: SOD rose while *CAT1* and *GPX2* were both suppressed, an effect the authors attributed to the resulting H_2_O_2_ accumulation, and zinc co-exposure restored *CAT1* and *GPX2* toward baseline, though not above it [18]. Our data diverge from this in one respect: *GPX1* in gill was not merely restored but substantially elevated above baseline, alongside SOD, which may reflect the difference between acute, controlled dosing and the chronic, naturally co-accumulated Zn/Cu burden studied here. This gill-specific rise in *GPX1*, without a matching rise in CAT, has a plausible biochemical basis: selenium-dependent glutathione peroxidases such as GPX1 remain catalytically effective at lower peroxide concentrations than catalase, which typically needs a near-saturating H_2_O_2_ pool to work efficiently [40]. The same asymmetry appeared at the pathway level: the peroxisome gene set, which houses catalase, was significantly downregulated in both tissues by GSEA, a signal ORA did not detect at all, though the hepatopancreas signal did not survive a stricter FDR threshold (Table S8). This matches the complete absence of any significant change in CAT activity or *CAT* expression measured by qPCR anywhere in our data.

Peroxide clearance in gill, then, shifted toward GPX1 rather than toward uniform induction of the whole antioxidant system. MDA nonetheless rose in both tissues, indicating that this shift did not fully keep pace with the added rate of superoxide dismutation. MDA is a general marker of lipid peroxidation; however, membrane lipid composition itself typically shifts over an animal’s growth and reproductive cycle. Some part of this rise may therefore reflect changing substrate availability for peroxidation rather than oxidative damage alone. All animals in this study were triploid, and gonad development in triploid *M. gigas* produced by tetraploid × diploid crosses is strongly reduced, though not fully abolished [41]. The reproductive component of such compositional change [36] is therefore likely to be smaller here than in diploid bivalves, but it cannot be excluded. The association between MDA and the SOD/CAT activity ratio likewise disappeared within sampling dates, indicating that it reflects the parallel rise in SOD activity and MDA over the culture period rather than a direct coupling between the two.

This gap between defense and damage was not spread evenly across the animal. Gill sits in continuous, direct contact with the surrounding water and is considered the primary site of both metal uptake and metal-driven physiological response in bivalves for this reason [34]. A divergence between gill and hepatopancreas in antioxidant and glutathione responses under metal exposure has been reported in the freshwater bivalve *Unio tumidus* [22]. *M. gigas* shows a comparable split specifically for metal handling: metallothionein induction rises in gill under metal exposure, whereas hepatopancreas instead loses total tissue protein under the same conditions. This indicates that the two tissues rely on different strategies rather than one response scaled down in the second [21]. Even within gill, the strategy for handling excess copper shifts with copper burden: metallothionein-like proteins account for a progressively smaller share of cytosolic copper binding as gill copper burden rises, with larger protein complexes taking on more of that role instead. This suggests that gill switches between metal-handling routes rather than relying on a single fixed one [42]. Our own data show a comparable asymmetry for the antioxidant response specifically: GPX activity and *GPX1* transcript rose in gill only, and *KEAP1* rose in gill only. Mapping the 20 candidate antioxidant genes onto their human orthologs in STRING returned twice as many significantly changed nodes in gill as in hepatopancreas (14 versus 7 of 18). Gill also showed pathway-level signatures that hepatopancreas did not share, with neuroactive ligand–receptor interaction and ECM–receptor interaction both significantly upregulated in gill alone, consistent with heightened intercellular communication and extracellular matrix remodeling in the tissue with direct environmental contact. Gill, in short, carried the great majority of the measurable oxidative and antioxidant response documented here, consistent with its established role as the tissue interface with the environment.

This gill-weighted picture does not hold for every downstream gene, however. *GSR*, *GGCT*, and the GST-family detoxification genes were downregulated in both tissues, not in gill alone, even though the antioxidant enzyme response above was almost entirely confined to gill. KEAP1 offers a partial explanation for this split. KEAP1 constitutively targets the transcription factor Nrf2 for ubiquitin-mediated degradation, and Nrf2, once stabilized, drives transcription from the antioxidant response element shared by a broad set of detoxification and glutathione-handling genes [43]. This axis is confirmed at the genomic level in *M. gigas*, and its experimental activation produces gill-weighted responses in the same direction reported here [15]. *KEAP1* itself was significantly upregulated in gill only, while *GSR*, *GGCT*, and the GST-family genes were coordinately downregulated in both tissues. The same pattern held at the pathway level, with glutathione metabolism, the peroxisome, and cytochrome P450-mediated xenobiotic metabolism all reaching significance as downregulated gene sets in both tissues, more strongly in gill. This leaves an apparent puzzle: *Cu/Zn-SOD* and *GPX1* are themselves commonly counted among Nrf2 target genes in other systems, yet both rose sharply in gill alongside, rather than in spite of, elevated *KEAP1*. Rather than undermining the KEAP1 account, this split is more consistent with the two responses having different upstream drivers within the same tissue: *KEAP1* upregulation plausibly suppresses Nrf2-dependent transcription of the GSH-cycling and detoxification genes, while *Cu/Zn-SOD* and *GPX1* induction is better explained by direct cofactor and substrate availability, potentially through metal-responsive transcription factors distinct from Nrf2, rather than by the Nrf2/KEAP1 axis itself. Adaptive transcriptional responses to stress are generally understood to combine a component shared across tissues or contexts with additional, more localized components layered on top of it [44]. The detoxification-gene pattern fits that structure: a baseline suppression shared by both tissues, with an additional, gill-specific component tied to locally elevated *KEAP1*. GSH content itself did not change significantly in either tissue, consistent with this being a transcriptional adjustment rather than a depletion of the GSH pool already present.

The reorganization in gill and hepatopancreas extends well beyond this detoxification program. Oxidative damage, specifically carbonylation of proteasome ATPase subunits, has been shown to directly impair the 26S proteasome’s capacity to degrade the protein substrates delivered to it. The 26S proteasome is, moreover, understood to be more vulnerable to inactivation by sustained oxidative stress than other components of the protein quality-control system [45,46]. A review of proteomic biomarkers in pollution-exposed bivalves reported a related pattern from a different angle, with reduced cellular defense proteins alongside increased protein degradation in mussels from a heavily polluted site [13]. In our data, protein ubiquitination and ubiquitin-protein transferase activity (GO) were the single most significantly upregulated GO terms in both gill and hepatopancreas, while the proteasome pathway (KEGG) and its core complex genes were significantly downregulated in both tissues over the same comparison. More protein, in other words, appears to have been tagged for degradation while the complex responsible for carrying out that degradation was itself constrained. This matches the proteasome-specific vulnerability to sustained oxidative stress described above, rather than a proteostasis system cycling normally. Ribosome biogenesis, translation, and spliceosome-related genes were also coordinately downregulated in both tissues, among the strongest and most consistent signals in our dataset. Lysosomal and phagocytic pathways were concurrently upregulated in both tissues, which may represent a compensatory clearance route operating alongside a proteasome under this kind of strain. Together, these findings point to a broader reallocation of cellular resources, away from new-protein-synthesis machinery and toward managing a proteostatic burden, over the course of the culture period.

Not every stock followed this same path, however. A genetic contribution to Zn/Cu accumulation capability itself has direct precedent in oysters. A quantitative genetic study of the congeneric *Magallana angulata* estimated moderate narrow-sense heritability for both Cu (0.189 ± 0.061) and Zn (0.373 ± 0.091) accumulation, while the genetic correlation between this accumulation capability and growth-related traits was consistently low. This indicates that families can differ in how they handle these two metals largely independently of how they differ in growth [47]. A genome-wide association study in the same species linked candidate zinc-transporter (ZIP family) loci to individual variation in Cd accumulation [31]. Functional follow-up on two of those candidates, *Zip1* and *Zip3*, showed that RNAi knockdown of the two paralogs shifted Cd uptake in opposite directions, demonstrating that closely related transport genes can be regulated in opposite directions within the same species [32]. A subsequent transcriptomic comparison of high- and low-accumulating individuals of this same species found significant differential expression of an antioxidant gene, *SOD3*, between the two phenotypic groups, indicating that antioxidant gene expression, and not only metal-transport gene expression, can differ systematically with accumulation phenotype in oysters [48]. In our own data, *Cu/Zn-SOD* and the gill-specific genes changed in the same direction across all three stocks, differing only in magnitude, but *GPX1* and *KEAP1* in hepatopancreas broke that pattern, with a significant stock × group interaction driven by genuine disagreement in direction across stocks. The most plausible reading is that this hepatopancreas divergence is a stock-specific regulatory difference in the same kind already documented for Zn/Cu handling in this genus, layered on top of the shared, growth-linked reprogramming described above rather than replacing it. With three commercial stocks rather than a designed genetic cross, however, this remains consistent with, rather than proof of, a genetic basis.

## 5. Conclusions

In this study, we integrated transcriptomics, enzyme assays, and qPCR to examine how growth and naturally accumulated Zn/Cu relate to antioxidant physiology in Pacific oysters across a full culture cycle. Growth, rather than metal burden itself, tracked the transcriptome-wide reprogramming observed here, which was broadest in gill but included upregulation of the *Cu/Zn-SOD* transcript and coordinated downregulation of detoxification and protein-synthesis machinery in both tissues. Of the four metals measured (Zn, Cu, Fe, and Mn), only Zn and Cu tracked growth in this way. This points to a metal-specific link rather than a generic consequence of filtering more water. Stock-level qPCR further showed that *GPX1* and *KEAP1* diverged in direction among stocks in hepatopancreas, one of the key sources of variability in this response. This appears to be the first pairing of naturally occurring, growth-period Zn/Cu accumulation with whole-transcriptome profiling in a bivalve. Together, these findings point to Zn/Cu bioaccumulation in this species as a marker of growth. The associated oxidative and proteostatic changes co-vary with growth and are consistent with being driven by growth physiology, rather than reflecting an independent, pollutant-specific signal.

## Data Availability

The raw transcriptome sequencing data (RNA-seq) generated in this study are openly available in the NCBI Sequence Read Archive (SRA) at https://www.ncbi.nlm.nih.gov/bioproject/PRJNA1501839 (accessed on 15 August 2026) under BioProject accession PRJNA1501839. All other data supporting the findings of this study are available within the article and its Supplementary Materials.

## Supplementary Materials

The following supporting information can be downloaded at https://www.mdpi.com/article/10.3390/antiox15081039/s1, Figure S1: Protein–protein interaction network of key antioxidant genes in gill and hepatopancreas; Figure S2: Top 10 most significantly up- and downregulated GO terms in gill and hepatopancreas; Figure S3: GSEA enrichment plots for glutathione metabolism, peroxisome, and metabolism of xenobiotics by cytochrome P450; Figure S4: Concordance between RNA-seq and qPCR log2 fold changes; Table S1: The primers used for qPCR; Table S2: Correlations between MDA content and SOD/CAT and SOD/GPX activity ratios; Table S3: Full differential expression results for the gill comparison (Hg vs. Lg); Table S4: Full differential expression results for the hepatopancreas comparison (Hh vs. Lh); Table S5: STRING protein–protein interaction network data; Table S6: Significantly enriched GO terms and KEGG pathways identified by over-representation analysis (ORA) in gill and hepatopancreas; Table S7: GSEA results for GO and KEGG gene sets in gill and hepatopancreas; Table S8: Sensitivity of the ORA–GSEA comparison to the GSEA significance threshold; Table S9: Per-stock qPCR relative expression data.
