# Supplementary material for: Growth-Linked, Tissue-Specific Antioxidant Reprogramming During Natural Zn/Cu Bioaccumulation in the Pacific Oyster Magallana gigas
Source: Antioxidants (Basel). 2026 Aug 21;15(8):1039. doi: 10.3390/antiox15081039 (PMC13509690; doi:10.3390/antiox15081039)
Supplement: Supplementary file 1 [file antioxidants-15-01039-s001.zip › Supplementary Figures.pdf]

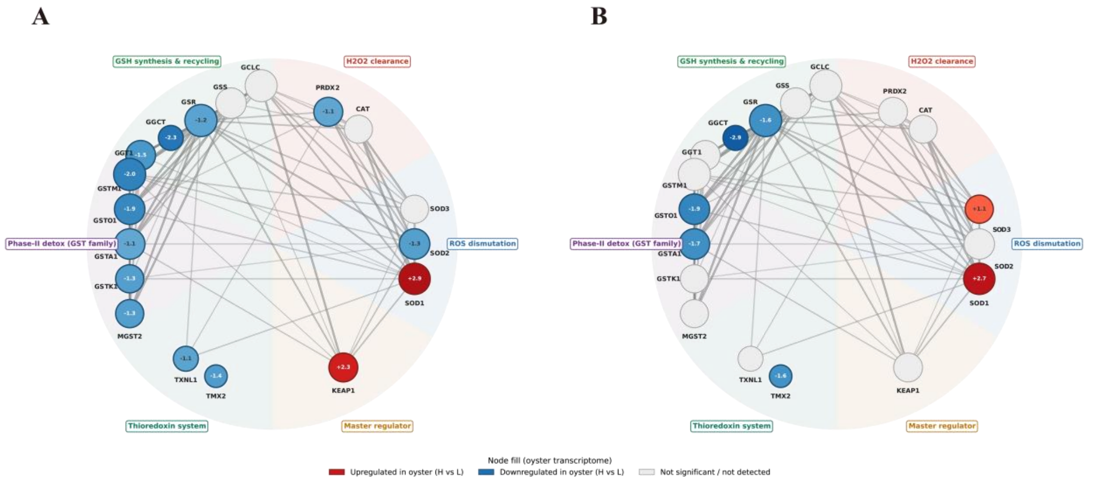

Figure S1. Protein–protein interaction network of key antioxidant genes: tissue-specific coverage. STRING (v12, human orthologs, confidence ≥ 0.4) network of the 18 antioxidant genes from Figure 4 with a STRING match, using an identical layout in both panels and grouped by functional category (colored labels). Node fill indicates the oyster log2FoldChange (H vs. L); grey indicates not significant/not detected. (A) Gill (Hg vs Lg). (B) Hepatopancreas (Hh vs Lh). Edges reflect known interactions among human orthologs (no *M. gigas*-specific interaction data exists in STRING).

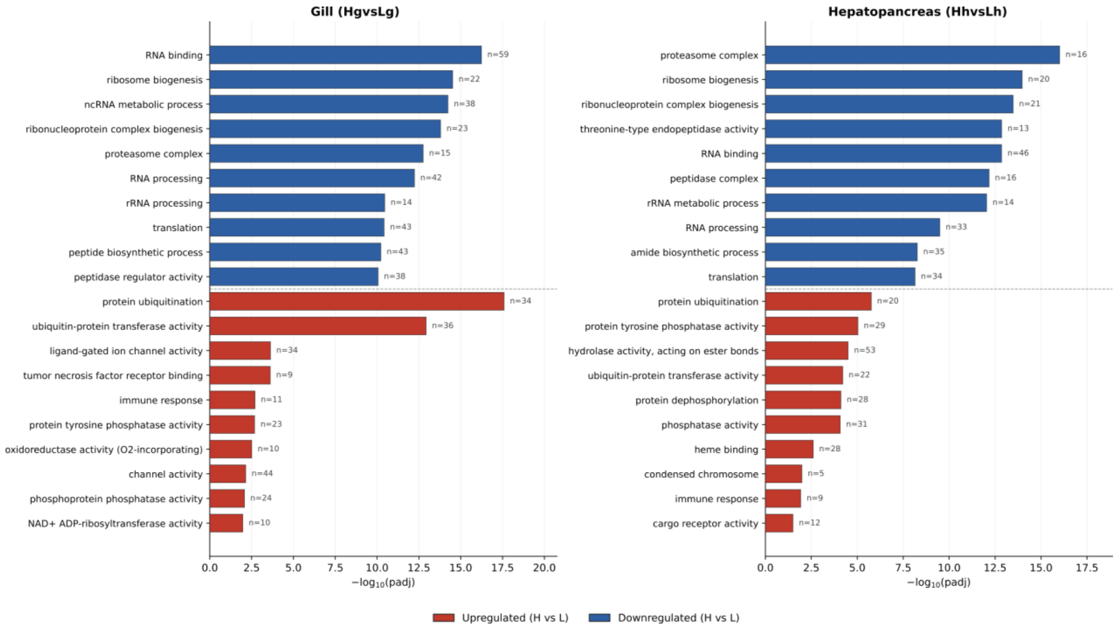

Figure S2. Top 10 most significantly upregulated and top 10 most significantly downregulated GO terms in gill (Hg vs Lg) and hepatopancreas (Hh vs Lh), ranked by adjusted p-value. Bar length reflects −log10(padj); the number of genes contributing to each term is shown alongside each bar. Red bars, upregulated (H vs L); blue bars, downregulated (H vs L).

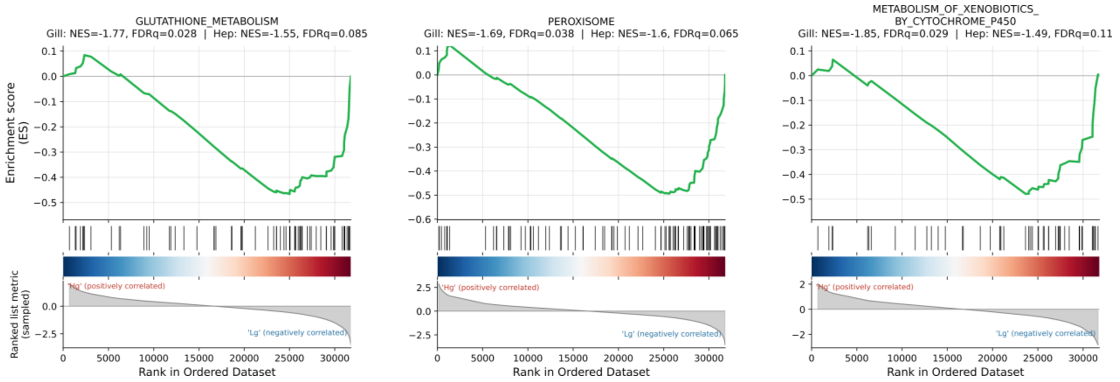

Figure S3. GSEA enrichment plots (gill, Hg vs Lg) for GLUTATHIONE_METABOLISM, PEROXISOME, and METABOLISM_OF_XENOBIOTICS_BY_CYTOCHROME_P450 (gene-set identifiers as given in MSigDB), showing the running enrichment score, gene-set member rank positions, phenotype-correlation color bar, and ranking-metric values sampled at gene-set member positions. NES and FDR q-values are shown for both gill and hepatopancreas above each panel.

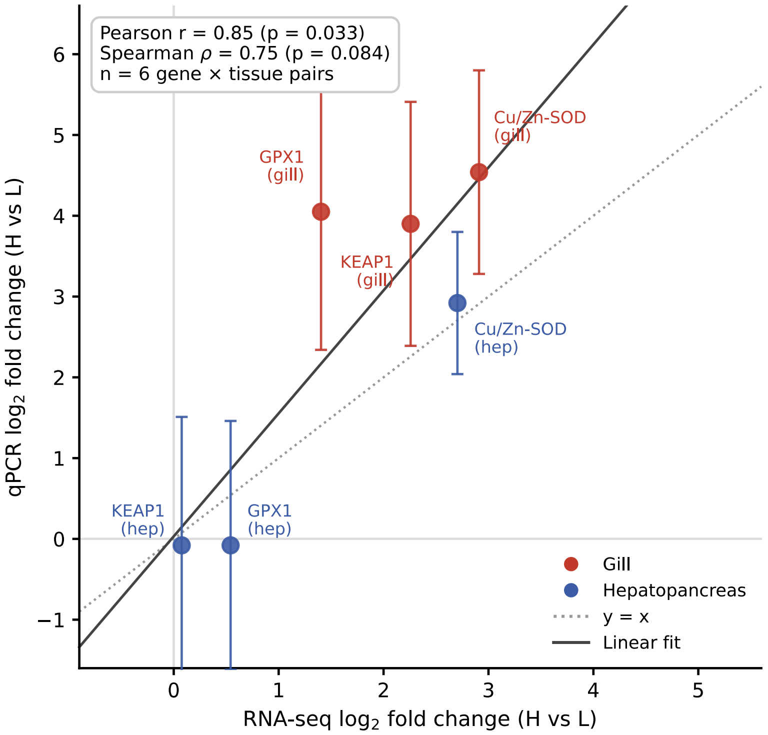

Figure S4. Concordance between RNA-seq and qPCR log2 fold changes (H vs L), assessed by Pearson and Spearman correlation across six gene × tissue pairs. Each point is one pair (gill, red; hepatopancreas, blue); error bars show the SD of qPCR log2 fold change across nine biological replicates. The dotted line is the identity line (y = x) and the solid line the least-squares fit. CAT is not included, as no catalase gene was present in the annotation used for read quantification and no RNA-seq log2 fold change is therefore available.
